# Sex Differences in Head Acceleration Events in Law Enforcement Corrections Cadets

**DOI:** 10.1007/s10439-025-03778-z

**Published:** 2025-06-20

**Authors:** Carly R. Smith, Enora Le Flao, Samantha N. DeAngleo, Jeffrey J. Wing, Nathan A. Edwards, James A. Onate, Joshua A. Hagen, Scott Paur, Joshua Walters, Jaclyn B. Caccese

**Affiliations:** 1 The Ohio State University Wexner Medical Center, College of Medicine, School of Health and Rehabilitation Sciences, Columbus, OH USA; 2 Chronic Brain Injury Program, The Ohio State University, Columbus, OH USA; 3 Human Performance Collaborative, The Ohio State University, Columbus, OH USA; 4 College of Public Health, The Ohio State University, Columbus, OH USA; 5 Franklin County Sheriff’s Office, Columbus, OH USA; 6 246 Atwell Hall, 453 W 10th Ave, Columbus, OH 43210 USA

**Keywords:** Repetitive head impacts, Concussion, Police, Combat training, Risk factors

## Abstract

**Purpose:**

Law enforcement cadets (LECs) undergo subject control technique training that may expose them to repetitive head impacts recorded as head acceleration events (HAEs) using instrumented mouthguards. Prior research suggests that sex and/or gender differences in HAE frequency and magnitude vary by sport. This study aimed to examine sex differences in HAE exposure among LECs during training.

**Methods:**

We collected HAEs from 82 civilian LECs (16 females, mean age = 30 ± 9 years) using instrumented mouthguards. We compared peak linear acceleration (PLA) and peak rotational velocity (PRV) of HAEs > 5 g between sexes using a mixed-effects linear model, with sex and cohort as fixed-effect predictors and a random intercept for subject to account for repeated HAEs within individuals. Additionally, we assessed sex differences in the number of HAEs per athlete exposure using a negative binomial regression controlling for cohort.

**Results:**

PLA was lower in female than male cadets (e.g., median PLA: females = 10.9 g, males = 12.3 g, p < 0.001). However, there were no statistically significant sex differences in the number of HAEs per athlete exposure (e.g., median: females = 10, males = 14, p = 0.169) or PRV (e.g., median PRV: females = 7.4 rad/s, males = 7.9 rad/s, p = 0.110).

**Conclusions:**

Overall, sex differences in HAE frequency and magnitude during subject control technique trainings were minimal. When differences were observed, female cadets exhibited less frequent and less severe HAEs than male cadets. This finding suggests that current training practices, including sex- and/or skill-matched pairing, may effectively reduce HAE exposure risk to females.

**Supplementary Information:**

The online version contains supplementary material available at 10.1007/s10439-025-03778-z.

## Introduction

Law enforcement cadets (LECs) undergo extensive combative training, known as “subject control technique training,” to prepare for encounters with suspects. This training includes defensive tactics such as Brazilian Jiu-Jitsu and boxing, exposing LECs to repetitive head impacts. These repetitive head impacts can result in head acceleration events (HAEs), that can be quantified in number and magnitude using instrumented mouthguards (IMGs), providing objective measures of exposure. Although most existing research focuses on sport-related HAEs [1–7], our landmark study demonstrated that LECs sustain a high frequency and magnitude of HAEs during training [8], comparable to levels observed in boxing [9–11].

Consistent with sport-related HAE studies, there is substantial variability in HAE exposure among LECs during training [8]. In sports, this variability has been attributed to several factors, including sex and/or gender differences (from here on referred to as “sex differences”) [10, 12–19]. In sports where men/boys and women/girls compete under different rules (e.g., ice hockey, lacrosse), differences in gameplay, regulations, and protective equipment may influence HAE exposure [15, 16]. However, even in sports with identical rules between genders (e.g., soccer), sex differences in HAE exposure persist [16, 20]. Although the underlying mechanisms remain unknown, contributing factors may include athlete size, strength, and style of play [21, 22]. Given these sport- and context-specific differences, examining sex differences in HAE exposure is critical for developing targeted injury risk reduction strategies and ensuring equitable safety measures across training and competition settings.

Although sex differences in HAEs have not been examined in occupational settings, combat sports provide a relevant comparative model [8]. Some combat sports enforce gender-based competition rules (e.g., in professional boxing, women compete in 10 two-minute rounds vs. men in 12 three-minute rounds), yet share similarities in equipment use and standardized rules (e.g., both men and women compete under the same regulations in mixed martial arts (MMA)) [23, 24]. Prior research on sport boxing training (sparring) reported that men sustained more HAEs than women, though HAE magnitudes were similar [25]. However, most combat sport literature either excludes women [10] or lacks a sufficient sample to examine sex differences [9, 26]. Subject control technique training for LECs is identical for men and women. This standardized training environment provides an opportunity to examine sex differences in HAE exposure. Determining whether male LECs sustain more HAEs than female LECs will help identify sex-specific risk factors in training and guide injury risk reduction efforts.

This fundamental subject control technique training, and the associated HAE exposures, cannot be eliminated. Therefore, it is critical to identify potential risk factors for higher exposure to develop targeted interventions that mitigate risk (e.g., individualized instruction for at-risk populations). Thus, this study aimed to determine sex differences in HAE frequency and magnitudes during subject control technique training. Based on prior research [25], we hypothesized that male LECs would sustain a higher number of HAEs per athlete exposure but with similar magnitudes to female LECs. A secondary objective was to assess data inclusion methods across LEC cohorts, given that video verification was only available for the final cohort. We hypothesized that HAE counts recorded during active training time and identified as true-positive by Prevent Biometrics (Active Time + PBTP) would not differ from the video-verified Prevent Biometrics true-positive HAEs (Video Verified + PBTP). Findings from this study will contribute to improving LEC health, safety, and well-being during mandatory training programs.

## Materials and Methods

### Participants

**Fig. 1:**
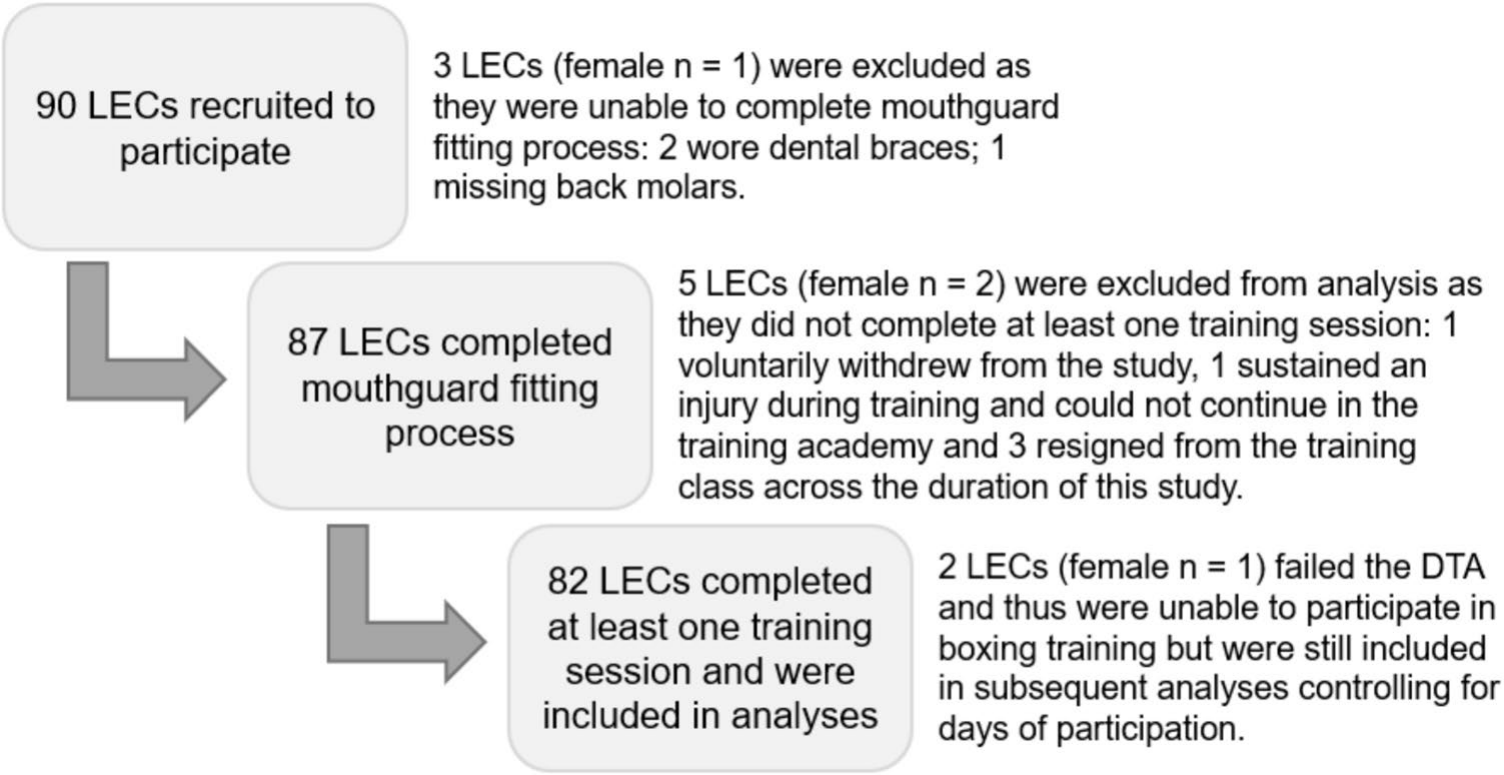
Participant consort diagram demonstrating the recruitment process and inclusion criteria for analyses

A total of 90 LECs from four corrections training academy cohorts (Fall 2022, Spring 2023, Fall 2023, and Winter 2023) were recruited for this study. LECs were excluded if a secure fit of the Prevent Biometrics (Edina, MN) mouthguard could not be achieved (n = 3; e.g., due to dental braces). Further detailed information on the mouthguard used in this study is provided below. No other exclusion criteria were applied to maximize the generalizability of our findings. Five LECs (including two women) were excluded from data analysis because they did not complete any training sessions, withdrew from the study, or resigned/were removed from the training academy (Fig. 1). Participants self-reported demographic information (Table 1). Prior to participation, all participants provided written informed consent that was approved by the University’s Institutional Review Board (Study ID 2022H0299). Data collection procedures are reported in accordance with the Consensus Head Acceleration Measurement Practices (CHAMP) 2022 checklist [27].

**Table 1:** Participant demographics and independent samples t-tests

	Total	Female	Male	Sex comparison (two-sided p)
Count (n)	82	16	66	
Age (years)	30 ± 9	29 ± 9	30 ± 9	0.692
Weight (kg)	91.2 ± 20.9	74.1 ± 18.8	95.4 ± 19.4	< 0.001
Height (m)	1.77 ± 0.09	1.65 ± 0.06	1.80 ± 0.07	< 0.001
Race				0.711
Asian	2.4% (2/82)	0% (0/16)	3.0% (2/66)	
Black or African American	13.4% (11/82)	12.5% (2/16)	13.6% (9/66)	
White	76.8% (63/82)	75% (12/16)	77.3% (51/66)	
More than 1 race	4.9% (4/82)	12.5% (2/16)	3.0% (2/66)	
Other	2.4% (2/82)	0% (0/16)	3.0% (2/66)	
Ethnicity				0.978
Hispanic	6.1% (5/82)	6.3% (1/16)	6.1% (4/66)	
Non-Hispanic	87.8% (72/82)	93.8% (15/16)	86.4% (57/66)	
Not reported	6.1% (5/82)	0% (0/16)	7.6% (5/66)	

### Data Collection Sessions

LECs completed six to seven sessions of subject control technique training, which included defensive tactics training (DT), a DT assessment (DTA), and boxing. All session were conducted under constant supervision by academy training staff. Additional details on the training academy structure have been previously published [8]. The number of sessions varied across cohorts due to changes in academy structure. Specifically, the first two cohorts completed 3 days of boxing 2 weeks after their DTA, whereas the last two cohorts completed two days of boxing prior to their DTA. Participant availability for each session also varied due to personal (e.g., injuries) and technological (e.g., battery failure, sensor malfunction) limitations. Our previous research [8] demonstrated that LECs experienced the highest frequency and severity of HAEs during DTA and boxing. Additionally, during DTA and boxing sessions, only one to two LECs were actively participating (i.e. exposed to head impacts) at a time. This setup allowed the research team to take precise timestamped notes of each participant’s active times, enhancing confidence in HAE inclusion, particularly since filming was not permitted for the first three cohorts. Thus, only HAEs recorded during DTA, and boxing sessions were included in these analyses. Although cumulative participation time was not recorded, each DTA session consisted of two, 2-min rounds of a simulated encounter with a training academy instructor acting as a “suspect,” and boxing sessions consisted of two to four sparring bouts lasting 2–3 min each. The average number of recorded sessions per participant (DTA and boxing combined) was 3.1 ± 0.9. A research team member was present at all data collection sessions and confirmed that all equipment was fully charged and functioning prior to each session.

### Instrumentation

This study used IMGs containing tri-axial accelerometers and gyroscopes to quantify HAEs. Participants were fitted with Prevent Biometrics’ boil-and-bite Impact Monitoring Mouthguard (Prevent Biometrics, Inc., Edina, MN) by a trained research team member following the manufacturer’s guidelines. At the time of fitting, a research team member confirmed that the IMG remained securely in place and did not become displaced from the upper dentition when participants opened their mouth.

**Fig. 2:**
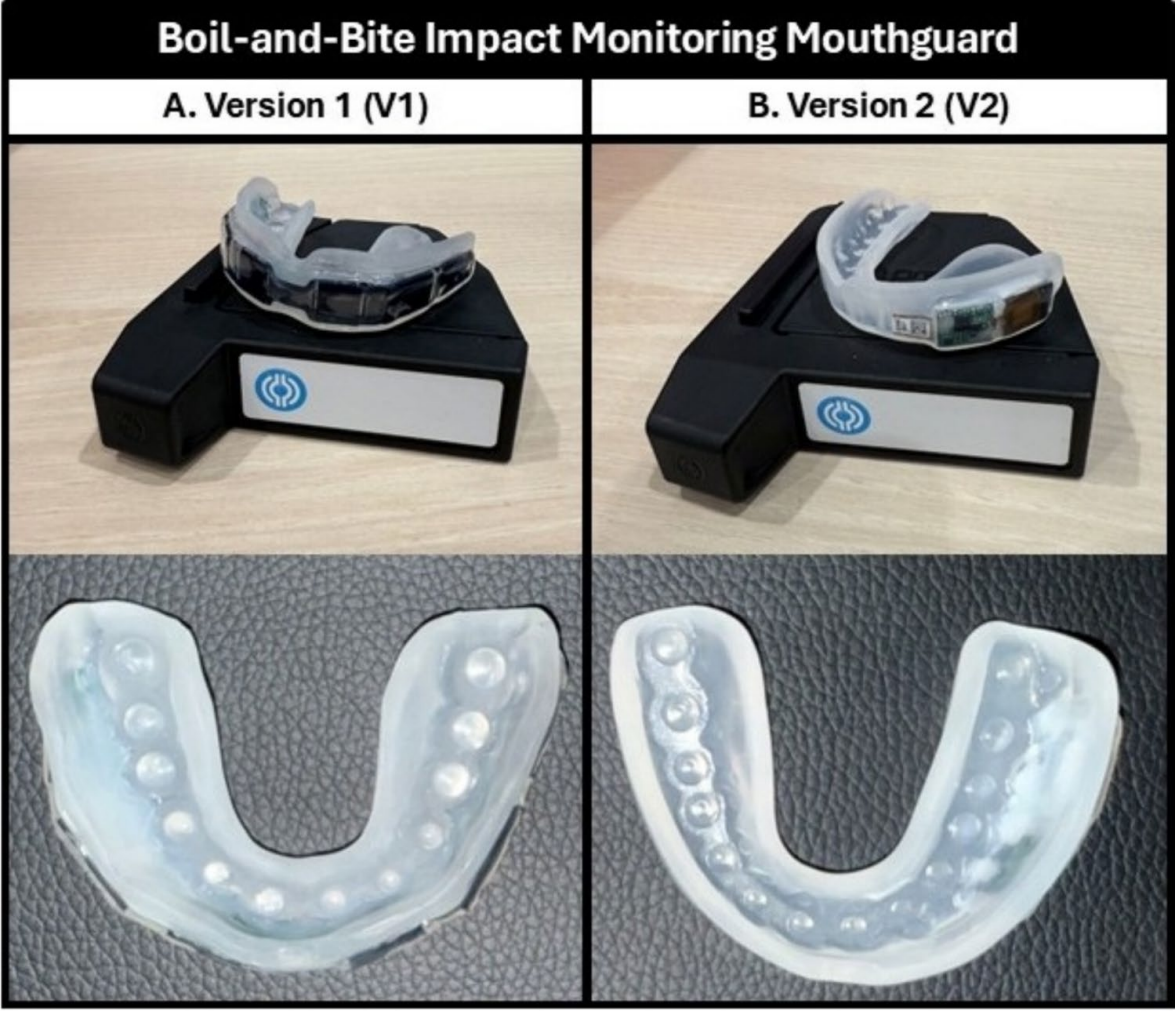
**A** Version 1 (V1) and **B** Version 2 (V2) of Prevent Biometrics’ boil-and-bite Impact Monitoring Mouthguard (Prevent Biometrics Inc., Edina, MN). V1 (used in Fall 2022 and Spring 2023) had sensors positioned fully around the IMG. V2 (used in Fall 2023 and Winter 2023) had smaller sensors located on only one side of the IMG

Over the course of the study, two versions of IMGs were used: Version 1 (V1) and Version 2 (V2) (Fig. 2). Although both versions featured identical sensor properties, their designs differed slightly. V1 (used in Fall 2022 and Spring 2023) had sensors positioned fully around the IMG. V2 (used in Fall 2023 and Winter 2023) had smaller sensors located on only one side of the IMG. The manufacturer’s proprietary post-processing algorithms were the same in both versions. To account for potential differences, cohort (and thus IMG version) was controlled for in all analyses.

The Prevent Biometrics Impact Monitoring Mouthguard measured linear acceleration via a tri-axial accelerometer up to 200 g and rotational velocity via a tri-axial gyroscope up to 35 rad/s, both sampling at 3200 Hz. These IMGs record data when linear acceleration exceeds 8 g in any axis. Post-processing (version 2.0.17) includes low-pass filtering (50–200 Hz) to reduce noise, which can attenuate peak values, particularly for high-frequency impacts. Each impact is assigned quality indicator (0–2, with 0 being the highest quality) based on the signal-to-noise ratio, which aimed to detect IMG movement relative to the teeth and other potential artifacts. The quality indicator determined the cut-off frequency used in low-pass filtering (0 = 200 Hz, 1 = 100 Hz, 2 = 50 Hz). A correction factor (ranging from 1.0 to 2.0) is then applied to the peak of the processed linear acceleration time series based on the degree of filtering. Therefore, the final PLA will be higher than the non-corrected peak and should better approximate the true peak. As a result, the final reported PLA values are estimates that may be slightly higher or lower than the true peak, as well as higher or lower than the raw data, depending on the extent of signal attenuation and the accuracy of the applied correction factor. Only events with a final PLA above 5 g after processing are retained. Although higher thresholds (10–15 g) are more common in sport-related HAE studies [28–30], a lower threshold (5 g) was chosen to capture more true-positive impacts. Because IMGs were worn only during active participation, and training occurred in a relatively controlled environment, motion artifacts from non-impact activities (e.g., running) were less of a concern compared to traditional sports settings [31–33].

Independent validation of the Prevent Biometrics’ boil-and-bite Impact Monitoring Mouthguard demonstrated that PLA (r^2^ = 0.99) and PRV (r^2^ = 0.92) were highly correlated with laboratory test devices across impact magnitudes of 25, 50, 75, and 100 g [34]. The mean relative error in peak magnitude was 2.5% for PLA and 4.6% for PRV [34].

### Event Verification and Data Inclusion Processes

Although video verification is the best practice for HAE monitoring [27], video recording was not permitted for the first three cohorts included in the analyses. Only the final cohort (Winter 2023) was video recorded as described below. To improve confidence in non-video-verified HAEs, we implemented multiple quality control measures. First, IMGs were worn only during active participation in training scenarios and removed during instructional time and rest breaks. Additionally, a research team member observed all training sessions, actively monitoring HAEs in real time and recording individual participation times. Because only DTA and boxing sessions were analyzed, a maximum of two LECs actively participating at any given time. This structure allowed for detailed tracking of participation times. Prior combat sports research using similar IMGs demonstrated that removing inactive time reduces false-positive rates to below 1% [9].

To further refine data accuracy, we leveraged Prevent Biometrics’ proprietary machine learning algorithm to filter out false-positive HAEs, referred to as “Active Time + PBTP”. Additionally, all HAE data from all four cohorts were downloaded on the same date to ensure consistency using the same algorithm version (2.0.17), as Prevent Biometrics periodically updated its software during the study. We also focused on high-quality HAEs classified as “0” (highest signal-to-noise ratio) by Prevent Biometrics, referred to as “Active Time + PBTP + 0”.

### Video Verification

The final cohort (Winter 2023) was video recorded during DTA and boxing sessions to enhance the accuracy of HAE identification. Due to space constraints, DTA was recorded from a single vantage point, while boxing sessions were recorded from two vantage points. The cameras (Sony Cyber-shot DSC-Rx100 V) recorded at 50 frames/s and a resolution of 1080 p. Following data collection, videos were synchronized using Hudl Sportscode (Agile Sports Technologies, Inc., USA). The timestamps of HAEs recorded by the IMGs were aligned with the videos using the world clock displayed in the recordings. One trained research team member conducted the video verification process for all recorded training sessions. HAEs were categorized as true-positive (i.e., LEC was actively participating in training, and a visualized impact occurred), unverifiable (i.e., LEC was actively participating, but the impact was not visible due to obstructions), false-positive (active no impact; i.e., LEC was actively participating but no visualized impact occurred), false-positive (inactive; i.e., LEC was not actively participating). The final Video Verified dataset was further refined to include only HAEs that were both video-verified as true-positive and classified as true-positive by Prevent Biometrics, resulting in the “Video Verified + PBTP” dataset.

### Statistical Analyses

#### HAE Sex Differences

First, we calculated descriptive statistics for all participants and also stratified by sex and data inclusion process. We used independent samples two-sided t-tests to compare demographic characteristics between sexes. To compare the number of HAEs per athlete exposure between sexes, we used negative binomial regression, controlling for training academy cohort to account for variations in total days of exposure and IMG version. To assess differences in PLA and PRV between sexes, we applied a mixed-effects linear model, with sex and cohort as fixed-effect predictors and a random intercept for subject to account for repeated HAEs within individuals. A Bonferroni correction was used to adjust for multiple comparisons across three outcome measures (HAEs per athlete exposure, PLA, PRV), resulting in an adjusted significance of ɑ = 0.05/3 = 0.017. These statistical analyses were estimated using SPSS statistical software (IBM SPSS Statistics for Windows, version 28.0.1.1. Armonk, NY: IBM Corp).

#### Measurement Quality and Consistency Across Inclusion Processes

To assess the impact of data inclusion methods on the results, all analyses were conducted using multiple datasets, including: “Active Time”, “Active Time + PBTP”, “Active Time + PBTP + 0”, “Video Verified”, and “Video Verified + PBTP”. Because only the final cohort (Winter 2023) underwent video verification, we acknowledge that the sample size for video-verified datasets is smaller compared to the other methods. To quantify differences in the number of HAEs identified across data inclusion processes, we calculated impact rate ratios (IRRs) and intraclass correlation coefficients (ICCs) using total HAE counts. IRRs have been used previously in HAE monitoring to compare the various, but commonly deployed, post-data collection cleaning techniques and assess the effect of these processes on final reported HAE characteristics (e.g., impact rates and magnitudes) [35]. This study calculated IRRs as the total number of HAEs recorded in one inclusion process divided by another inclusion process (e.g., IRR = HAEs in Active Time/HAEs in Active Time + PBTP = 375 HAEs/332 HAEs = 1.13). Because video verification was only available for Winter 2023 participants (n = 13), ICC and IRR calculations were performed using only HAEs recorded from this subset, ensuring all five data inclusion methods were represented in these analyses. These statistical analyses were estimated using Microsoft Excel (Microsoft 365, Version 2501; Redmond, Washington).

## Results

### Assessment of Data Inclusion Methods

**Fig. 3:**
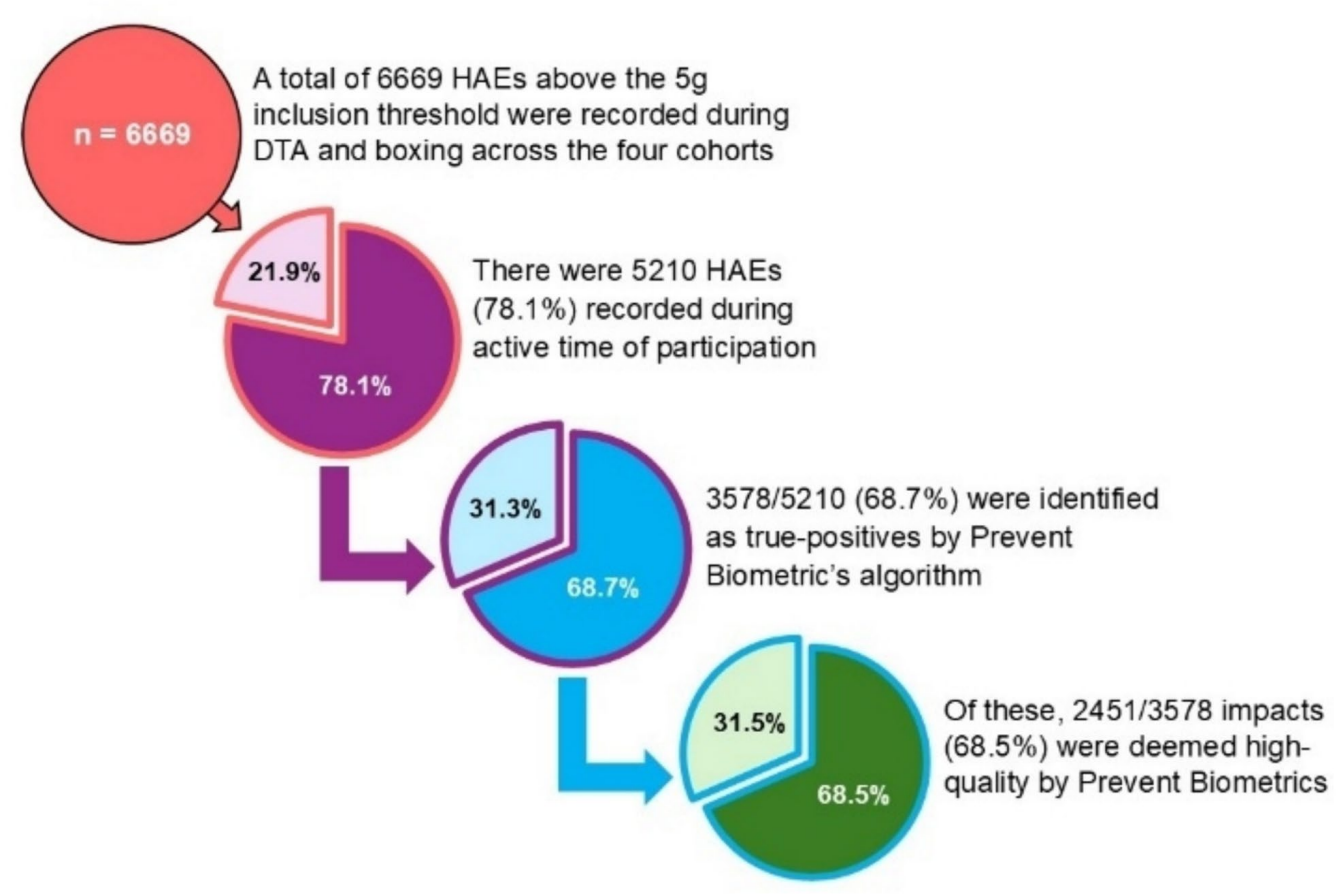
Depiction HAEs included in the assessment of data inclusion methods for all cohorts

A total of 6669 HAEs above the 5 g inclusion threshold were recorded during DTA and boxing across the four cohorts. There were 5210 HAEs (78.1%) recorded during active participation, of which 3578/5210 (68.7%) were classified as true-positive HAEs by Prevent Biometrics’ algorithm. Among the true-positive HAEs, 2451/3578 impacts (68.5%) were deemed high-quality by Prevent Biometrics (Fig. 3).

**Fig. 4:**
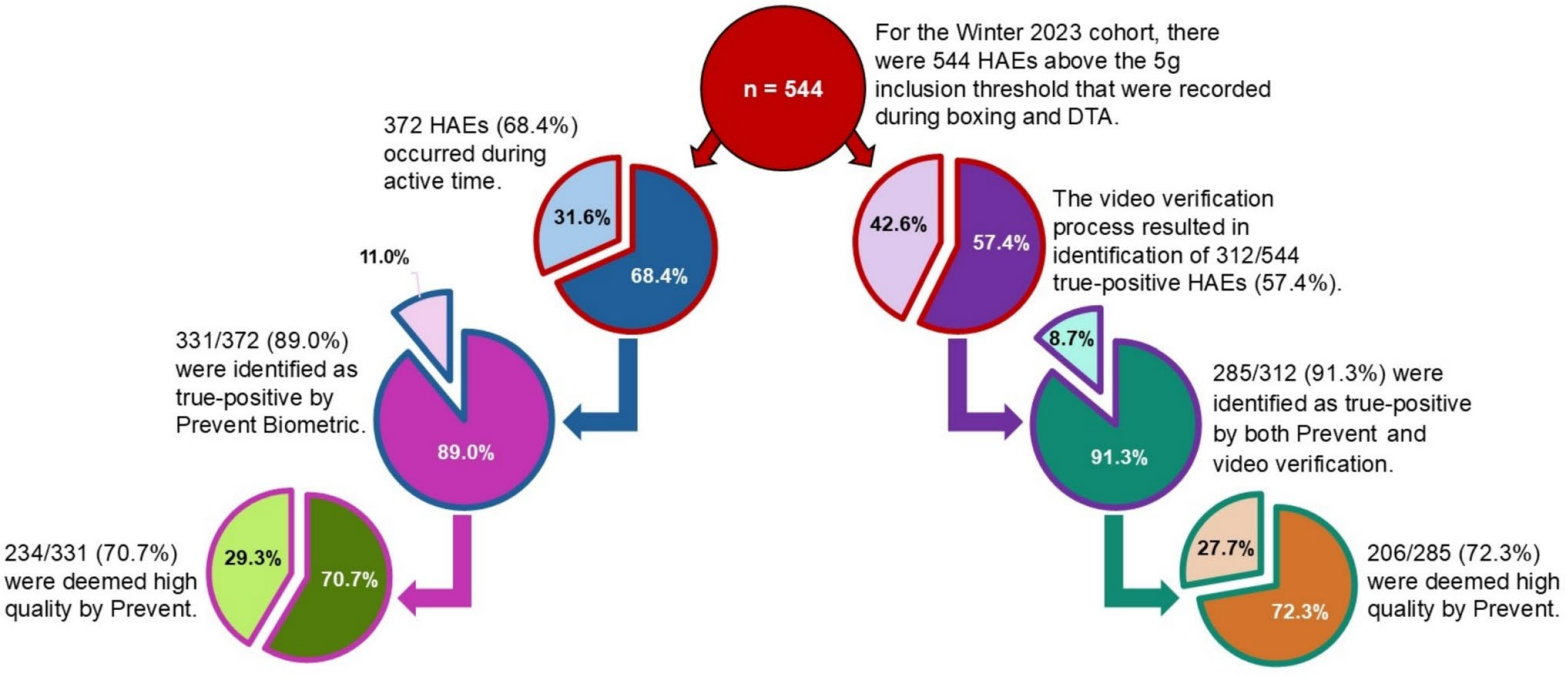
Depiction HAEs included in the assessment of data inclusion methods for Winter 2023 cohort

For the Winter 2023 cohort, there were 544 HAEs above the 5 g inclusion threshold that were recorded during boxing and DTA. There were 372 HAEs (68.4%) recorded during active participation, of which 331/372 (89.0%) were classified as true-positive HAEs by Prevent Biometrics’ algorithm. Of these, 234/331 (70.7%) were deemed high-quality by Prevent Biometrics. The video verification process identified 312/544 HAEs (57.4%) as true positives. Among these, 285/312 (91.3%) were classified as true positives by both Prevent Biometrics and video verification and 206/285 (72.3%) were deemed high-quality by Prevent Biometrics (Fig. 4).

Significant differences in IRRs were observed between “Active Time”, “Active Time + PBTP”, and “Active Time + PBTP + 0” compared to some of our other data inclusion methods. However, no significant differences in IRRs were observed between “Active Time + PBTP”, “Video Verified”, and “Video Verified + PBTP” (Table 2). ICCs between inclusion processes ranged from good (0.75 ≤ ICC < 0.9) to excellent (ICC ≥ 0.9) reliability, with “Active Time”, “Active Time + PBTP”, “Video Verified” demonstrating excellent reliability compared to the current best practice “Video Verified + PBTP” (ICC ≥ 0.9, Table 2).

**Table 2:** Impact rate ratios (IRRs) and intraclass correlation coefficients (ICCs) for Winter 2023 Cohort (n = 13 LECs), presented as IRR (95% confidence interval [CI]; ICC) comparing column process to row process

Data inclusion process (HAE n = )	Active Time	Active Time + PBTP	Active Time + PBTP + “0”	Video Verified	Video Verified + PBTP
Active Time (375)	–	IRR = 1.13 (0.97 to 1.31); ICC = 0.972***	IRR = 1.60 (1.36 to 1.89)^†^; ICC = 0.792**	1.20 (1.03 to 1.40)^†^; ICC = 0.978***	1.32 (1.13 to 1.53)^†^; ICC = 0.969***
Active Time + PBTP (332)		–	1.42 (1.20 to 1.68)^†^; ICC = 0.854**	1.06 (0.91 to 1.24); ICC = 0.967***	1.16 (0.99 to 1.36); ICC = 0.981***
Active Time + PBTP + 0 (234)			–	0.75 (0.63 to 0.89)^†^; ICC = 0.791**	0.82 (0.69 to 0.98)^†^; ICC = 0.829**
Video Verified (312)				–	1.09 (0.93 to 1.29); ICC = 0.991***
Video Verified + PBTP (285)					–

If 1.00 is contained within CIs, no significant differences; if 1.00 is not within the CI, there is a significant difference^†^. **Good reliability (0.75 ≤ ICC < 0.9); ***Excellent reliability (ICC ≥ 0.9). Prevent Biometrics designated true-positive head impact (PBTP); head acceleration event (HAE); interquartile ranges (IQR)

### Descriptive Statistics

There were 11 females and 42 males who used iMG V1, and 5 and 24, respectively, for V2. Descriptive statistics of HAEs, including means and standard deviations and medians and interquartile ranges, are reported overall, by sex, and by data inclusion processes for each outcome measure (Tables 3, 4, 5). These data are also depicted in violin plots provided in Supplementary Material.

**Table 3:** Head acceleration events per athlete exposure descriptive outcomes, presented as mean ± standard deviation and median (interquartile range), and comparison between sexes and effect of cohort for all cohorts (n = 82 LECs) and Winter 2023 cohort (n = 13 LECs)

Data inclusion process (HAE n = )	Total		Female		Male		Sex effect on HAE exposure (male as referent)			Cohort effect on HAEs
	Mean ± SD	Median (IQR)	Mean ± SD	Median (IQR)	Mean ± SD	Median (IQR)	Exp(β)	95% CI for Exp(β)	p-value	Type III p-value
All cohorts (HAE n = 6669)										
Active Time (5210)	19 ± 11	18 (12 to 24)	14 ± 11	12 (8 to 18)	21 ± 10	20 (14 to 25)	0.767	0.419 to 1.404	0.389	0.261
Active Time + PBTP (3578)	14 ± 8	13 (7 to 19)	9 ± 5	10 (4 to 13)	15 ± 9	14 (8 to 20)	0.647	0.348 to 1.203	0.169	0.784
Active Time + PBTP + “0” (2451)	10 ± 7	9 (4 to 13)	6 ± 4	7 (2 to 10)	10 ± 8	9 (5 to 15)	0.694	0.370 to 1.304	0.257	0.182
Winter 2023 cohort (HAE n = 544)										
Video Verified (313)	9 ± 5	8 (6 to 11)	6 ± 4	5 (5 to 8)	11 ± 5	9 (8 to 13)	0.610	0.186 to 2.002	0.415	–
Video Verified + PBTP (285)	8 ± 5	8 (5 to 10)	6 ± 4	4 (4 to 8)	9 ± 4	8 (7 to 11)	0.619	0.187 to 2.047	0.432	–

Prevent Biometrics designated true-positive head impact (PBTP); head acceleration event (HAE); interquartile ranges (IQR), exponentiated coefficient [Exp(β)]; confidence interval (CI); standard deviations (SD); ɑ = 0.017

**Table 4:** Peak linear acceleration (PLA, g) descriptive outcomes, presented as mean ± standard deviation and median (interquartile range), and comparison between sexes and effect of cohort for all cohorts (n = 82 LECs) and Winter 2023 cohort (n = 13 LECs)

Data inclusion process (HAE n = )	Total		Female		Male		Sex effect on PLA (male as referent)			Cohort effect on PLA
	Mean ± SD	Median (IQR)	Mean ± SD	Median (IQR)	Mean ± SD	Median (IQR)	β	95% CI	p-value	Type III p-value
All cohorts (HAE n = 6669)										
Active Time (5210)	14.7 ± 8.6	12.3 (8.9 to 18.1)	13.8 ± 7.7	11.6 (8.5 to 16.6)	14.9 ± 8.8	12.4 (8.9 to 18.4)	− 1.078	− 1.822 to − 0.335	0.004*	< 0.001*
Active Time + PBTP (3578)	14.5 ± 8.2	12.1 (8.9 to 17.6)	12.3 ± 5.8	10.9 (8.2 to 14.5)	14.7 ± 8.4	12.3 (9.0 to 18.1)	− 2.453	− 3.336 to − 1.571	< 0.001*	< 0.001*
Active Time + PBTP + “0” (2451)	13.3 ± 7.0	11.5 (8.6 to 16.0)	11.3 ± 4.5	10.6 (8.0 to 13.5)	13.6 ± 7.3	11.6 (8.7 to 16.4)	− 2.097	− 2.990 to − 1.203	< 0.001*	0.005*
Winter 2023 cohort (HAE n = 544)										
Video Verified (313)	13.9 ± 8.1	11.6 (9.1 to 16.2)	12.1 ± 5.3	11.0 (8.4 to 14.1)	14.5 ± 8.7	12.3 (9.3 to 17.4)	− 2.442	− 4.534 to − 0.350	0.022	–
Video Verified + PBTP (285)	13.4 ± 6.9	11.5 (9.0 to 15.6)	11.9 ± 5.1	11.0 (8.7 to 14.0)	13.9 ± 7.4	12.1 (9.1 to 16.7)	− 1.979	− 3.847 to − 0.111	0.038	–

PBTP, Prevent Biometrics designated true-positive head impact; head impact (PBTP); head acceleration event (HAE); interquartile ranges (IQR), exponentiated coefficient [Exp(β)]; confidence interval (CI); standard deviations (SD); ɑ = 0.017.

**Table 5:** Peak rotational velocity (PRV, rad/s) descriptive outcomes, presented as mean ± standard deviation and median (interquartile range), and comparison between sexes and effect of cohort for all cohorts (n = 82 LECs) and Winter 2023 cohort (n = 13 LECs)

Data inclusion process (HAE n = )	Total		Female		Male		Sex effect on PRV (Male as referent)			Cohort effect on PRV
	Mean ± SD	Median (IQR)	Mean ± SD	Median (IQR)	Mean ± SD	Median (IQR)	β	95% CI	p-value	Type III p-value
All cohorts (HAE n = 6669)										
Active Time (5210)	8.7 ± 5.1	7.6 (5.0 to 11.3)	8.4 ± 4.9	7.4 (5.1 to 10.4)	8.7 ± 5.2	7.6 (4.9 to 11.4)	− 0.293	− 0.737 to 0.150	0.195	0.452
Active Time + PBTP (3578)	8.7 ± 4.8	7.8 (5.2 to 11.3)	8.2 ± 4.2	7.4 (5.3 to 10.3)	8.8 ± 4.9	7.9 (5.2 to 11.4)	− 0.424	− 0.944 to 0.095	0.110	0.008*
Active Time + PBTP + “0” (2451)	8.7 ± 4.5	7.8 (5.3 to 11.3)	7.8 ± 3.8	7.1 (5.0 to 9.9)	8.8 ± 4.6	7.9 (5.4 to 11.4)	− 0.640	− 1.217 to − 0.062	0.030	< 0.001*
Winter 2023 cohort (HAE n = 544)										
Video Verified (313)	8.9 ± 5.5	7.8 (5.2 to 10.7)	8.9 ± 4.4	8.1 (5.7 to 11.6)	8.8 ± 5.8	7.5 (5.0 to 10.5)	0.062	− 1.369 to 1.493	0.932	–
Video Verified + PBTP (285)	8.5 ± 4.6	7.6 (5.3 to 10.5)	8.9 ± 4.0	8.1 (5.8 to 11.5)	8.3 ± 4.8	7.4 (5.0 to 9.9)	0.506	− 0.748 to 1.759	0.428	–

PBTP, Prevent Biometrics designated true-positive head impact; head impact (PBTP); head acceleration event (HAE); interquartile ranges (IQR), exponentiated coefficient [Exp(β)]; confidence interval (CI); standard deviations (SD); ɑ = 0.017

### HAEs Per Athlete Exposure

On average, females sustained fewer HAEs per athlete exposure than male LECs, though this difference was not significantly significant (p = 0.169–0.432; Table 3). No significant cohort differences were observed for HAEs per athlete exposure, and results were consistent across all data inclusion processes.

### PLA

Significant sex differences were observed for most data inclusion methods, with female LECs exhibiting lower PLA than male LECs (Table 4). However, when restricting the analysis to “Video Verified” and “Video Verified + PBTP” methods (single cohort only), sex differences were not statistically significant (p = 0.022 and 0.038, respectively; Table 4). Cohort differences across data inclusion methods were also significant (Table 4). Pairwise comparisons (Active Time + PBTP + 0) showed that relative to Winter 2023 cohort, there was between 0.76- and 1.63-units higher PLA across cohorts, with Fall 2022 having the highest PLA (p = 0.001).

### PRV

No significant sex differences were observed for PRV (Table 5). Cohort differences were observed for “Active Time + PBTP” and “Active Time + PBTP + 0” inclusion methods (p < 0.001 and 0.008, respectively; Table 5). Pairwise comparisons (Active Time + PBTP + 0) showed that relative to Winter 2023 cohort, there was between 0.42- and 1.60-units higher PRV across cohorts, with Spring 2023 having the highest PRV (p < 0.001).

## Discussion

The purpose of this study was to determine sex differences in the frequency and magnitude of HAEs sustained by LECs during subject control technique training. We hypothesized that male LECs would sustain a higher number of HAEs per athlete exposure than female LECs, with similar magnitudes. Contrary to our hypotheses, there were no significant sex differences in HAE exposure rates, and female LECs sustained significantly lower PLA compared to male LECs.

Overall, our findings provide limited evidence of sex differences in HAE exposure during LEC training. The lack of statistically significant differences in HAEs per athlete exposure between male and female LECs may be attributed to the structured nature of training, where all participants were required to complete the same sessions for the same duration. This differs from other studies on combat sports (e.g., boxing), where participants could self-select effort levels and duration of engagement [9, 25]. As there is limited work in occupational combat HAE exposure, comparisons to sport combat are often drawn. This highlights the importance of scenario/sport-specific research to make evidence-informed recommendations. Despite the lack of statistical significance, a clinically meaningful difference may still exist. Female LECs sustained anywhere from one-half to two-thirds as many HAEs per athlete exposure compared to their male counterparts. Our small sample size and high variability may have limited our ability to detect statistical significance, and additional factors such as participant size, combat sport experience, or competitiveness could also contribute to exposure differences.

Although PLA was significantly lower in female LECs, the difference was relatively small (1–2 g) and may not be clinically meaningful. Training instructors matched LECs for boxing training based on sex, height, weight, and perceived skill level, ensuring comparable pairings. These findings suggest that current training practices, particularly sex- and skill-matched pairings, may effectively mitigate sex-related disparities in HAE exposure. Because sex-matched pairing is not a standardized requirement across all law enforcement agencies, our findings suggest that implementing such practices could help reduce frequency and severity of HAEs across LECs. However, we acknowledge that using these practices may not accurately replicate the “real-world” scenarios that LECs are training for, such as managing encounters with inmates/suspects who are of different sex, size, and/or skill than themselves. In addition to trainings as described above, the training academy also includes non-contact “walk through” training exercises. During these exercises LECs act out the skills being taught with random partner pairings (i.e., not selected by academy trainers), safely exposing them to applying DT skills learned across various scenarios. Consistent with training academy leadership, we feel that this may be an appropriate way to expose LECs to more “real-world” scenarios without unnecessarily subjecting them to additional HAE exposure or injury risk. Additionally, there is a need for further research into other risk and protective factors that may influence HAE exposure. For example, modifiable factors such as participant strength and size [6, 36–38] and non-modifiable factors like prior combat sport experience [39] should be examined.

Reducing unnecessary HAE exposure would benefit all LECs, as they sustain a high rate of HAEs per training session. On average, LECs sustain 14 HAEs per athlete exposure, with individual exposures lasting as little as 4 min (DTA: two, 2-min rounds or up to 10–15 min; boxing: two to four 1–3-min sparring rounds). This exposure rate is comparable to combat sports such as boxing, where athletes sustain 5 to 50 HAEs per training session (average session duration ~ 25 min), with reported impact rates of 2–4 HAEs per minute [9, 10, 25]. In contrast, traditional contact sports such as amateur Australian football (1–3 HAEs per athlete exposure [40]), youth football (6–8 HAEs per athlete exposure [41–43]), soccer and ice hockey (1–3 HAEs per athlete exposure [20, 44]) report fewer HAEs per exposure than LECs. Yet, LECs have less access to healthcare providers when compared to traditional sports and fewer evidence-based injury risk reduction strategies. This reduced access to care may be attributed to the culture of law enforcement personnel not wanting to seek help, or due to difficulty in receiving the care (e.g., no healthcare professionals on site, therefore having to seek care off-hours) [45, 46]. In law enforcement personnel, nearly 80% of all reported head injuries were undiagnosed and/or untreated (1380/1799) [47], compared to 30–60% in traditional sports [48]. Improving access to healthcare professionals during and after training is critical. Athletic trainers and sports medicine professionals are commonly present during sports practices and could be integrated into LEC training settings to provide immediate care and enhance injury prevention efforts [49]. In high school football, reduced full-contact practice regulations successfully decreased HAE exposures [50]. Similarly, continued HAE monitoring in law enforcement training can guide injury risk reduction protocols, using frameworks established in sports medicine but tailored for occupational training.

LECs were instructed to limit sparring intensity to less than 50% of their maximum effort, but anecdotal evidence from the completion of this study suggests that some exceeded this threshold. Training academy leaders and research team members on-site during data collections noted multiple instances of LECs increasing their intensity, some of which subjectively exceeded the 50% effort threshold. Consistent with rule modifications in football to limit high-impact drills, training academy instructors could increase oversight of participant effort levels to ensure adherence to recommended intensity level. This would preserve skill acquisition while reducing unnecessary HAEs.

To assess the effects of data inclusion methods, we conducted analyses using five data inclusion methods, or “data cleaning methods”, commonly used in HAE monitoring studies. Findings on sex differences were largely consistent across inclusion methods, with minor variations (e.g., video verification methods showing no significant sex effect on PLA), likely due to smaller sample sizes in video-verified datasets. Our primary discussion is based on findings of “Active time + PBTP”, as this method included all four cohorts and has been used in prior research [8]. Although video verification is considered a best practice, we leveraged this study as an opportunity to assess whether “Active Time + PBTP” was a valid proxy for video verification. Because many impacts in combat sports occur directly to the mouth area, it is feasible that the IMG may become dislodged, potentially leading to noisy signals and the Prevent algorithm classifying such HAEs as false positives. Therefore, it is crucial to exclude HAE severity measures when the IMG became dislodged, as they may not accurately reflect true PLA/PRV and could lead to erroneous conclusions about impact magnitude. Relative to the data inclusion process used in our primary analyses (i.e., “Active Time + PBTP”), “Video Verified” (ICC = 0.967) and “Video Verified + PBTP” (ICC = 0.981) demonstrated excellent reliability (i.e., ICC ≥ 0.9). These findings suggest that the outcomes of these data inclusion processes are closely matched. When comparing the IRRs, or the agreement in the number of HAEs reported, of “Video Verified” and “Video Verified + PBTP” to “Active Time + PBTP” (i.e., data inclusion process used for this study’s primary analyses), we see that there were no significant differences between “Video Verified” (IRR = 1.06; 95% CI 0.91–1.24) or “Video Verified + PBTP” (IRR = 1.16; 95% CI 0.99–1.36). Thus, it would seem that a best practice approach would be to analyze HAE count from “Video Verified” but PLA/PRV from “Video Verified + PBTP”. For controlled settings as in this study, “Active time + PBTP” may be a valid proxy for video verification. Of note, we believe the apparent discrepancy in true positive HAE rate for the overall data (68.7%) and the Winter 2023 cohort (89.0%) is due to that the way we took timestamps became more precise as we went through the cohorts (e.g., cohort 1 was active time “1 to 4 pm” and in later cohorts, active time was noted “2:14:05 to 2:15:10”). Our more precise time-windowing in Winter 2023 would have eliminated a lot of false positives, and the proportion to true positives would be higher.

This study had several limitations. First, all participants were recruited from a single training academy, so our findings may not be generalizable to other training academies that use different training structures. Second, we included multiple cohorts, which varied in IMG version, instruction style, training intensity, and participant effort. However, cohort was controlled for in all analyses to mitigate these effects. Despite effect of cohort being significant in the overall model for some data inclusion processes, results from pairwise comparisons demonstrated no consistent pattern that would indicate IMG version differences that were not accounted for in controlling for cohort (e.g., if Fall 2022 and Spring 2023 had significantly higher PLAs than Winter 2023 and Fall 2023 did not). These differences may be attributed to the change from the first two cohorts completing 3 days of boxing 2 weeks after their DTA, whereas the last two cohorts completed only 2 days of boxing prior to their DTA. Third, HAEs recorded in this study may not reflect real-world law enforcement conditions, where HAEs may be more severe. Finally, sex was self-reported, but findings may reflect both sex and/or gender differences.

Identifying potential injury risk and protective factors is critical for developing risk reduction strategies specific to LEC training. Although LECs train under identical conditions sex-matched, size-matched, and skill-matched pairings appear to be effective in mitigating sex-related differences in HAE exposure. Expanding similar pairing practices across other law enforcement agencies may further reduce unnecessary HAEs. Future research should explore other risk and protective factors in LECs training to promote the health and well-being of LECs during their participation in mandatory training prior to beginning their law enforcement careers.

## Supplementary Information

Below is the link to the electronic supplementary material.

Supplementary file1 (DOCX 844 KB)

## Data Availability

Datasets generated and analyzed during the current study will be available in the FITBIR repository (https://fitbir.nih.gov/).

## Code Availability

Not applicable.
