# Supplementary material for: Sex Differences in Head Acceleration Events in Law Enforcement Corrections Cadets
Source: Ann Biomed Eng. 2025 Jun 20;53(9):2251–63. doi: 10.1007/s10439-025-03778-z (PMC12391156; doi:10.1007/s10439-025-03778-z)
Supplement: Supplementary file 1 — Supplementary file1 (DOCX 844 KB) [file 10439_2025_3778_MOESM1_ESM.docx]

**Supplementary Materials**


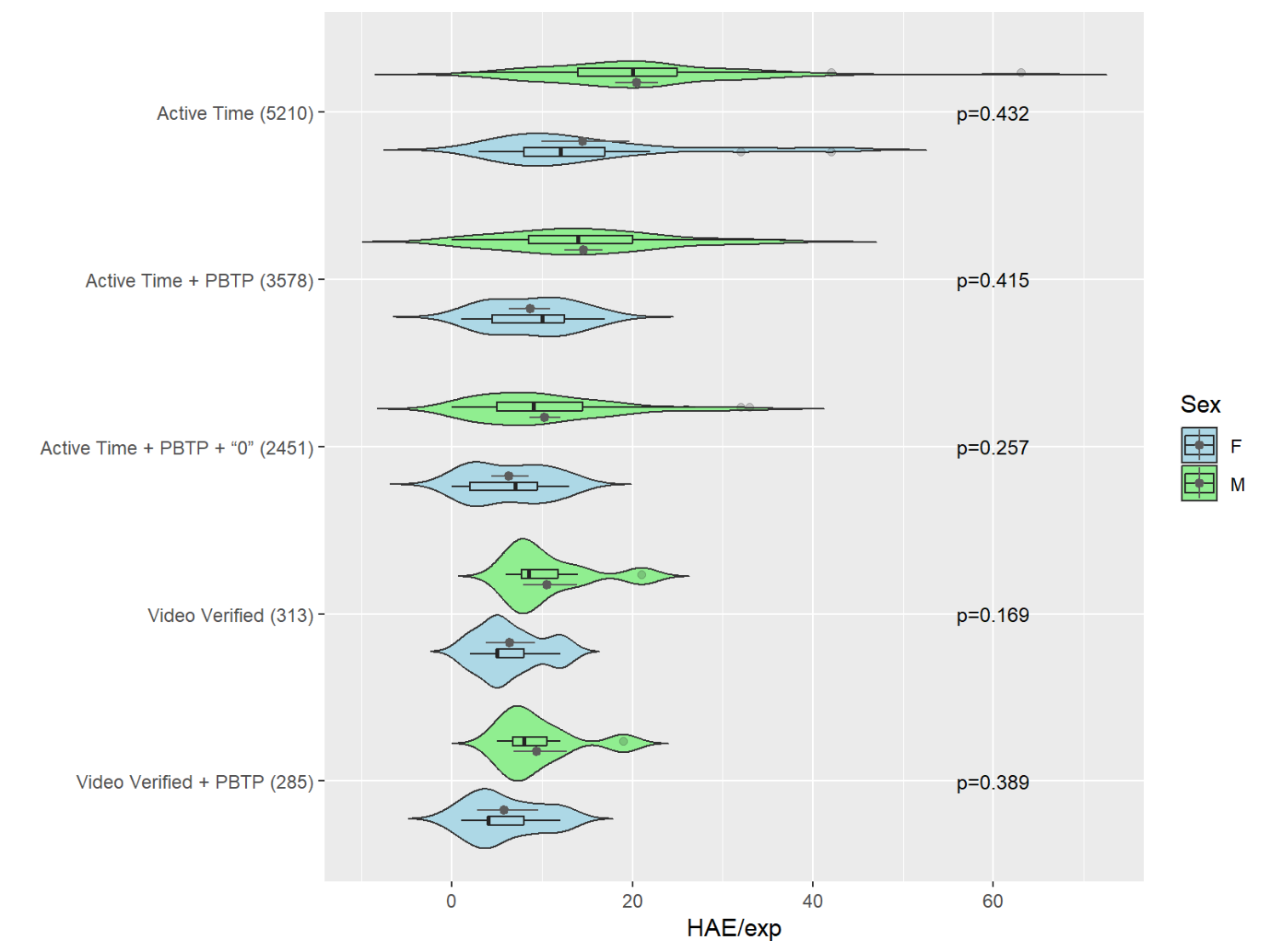


**Supplementary Figure 1.** Violin plots of Head Acceleration Events per Athlete Exposure between sexes. Boxplots within the violins represent the median and interquartile range; point with line represents the mean +/- standard deviation; p-value corresponds to the sex difference within data inclusion process.


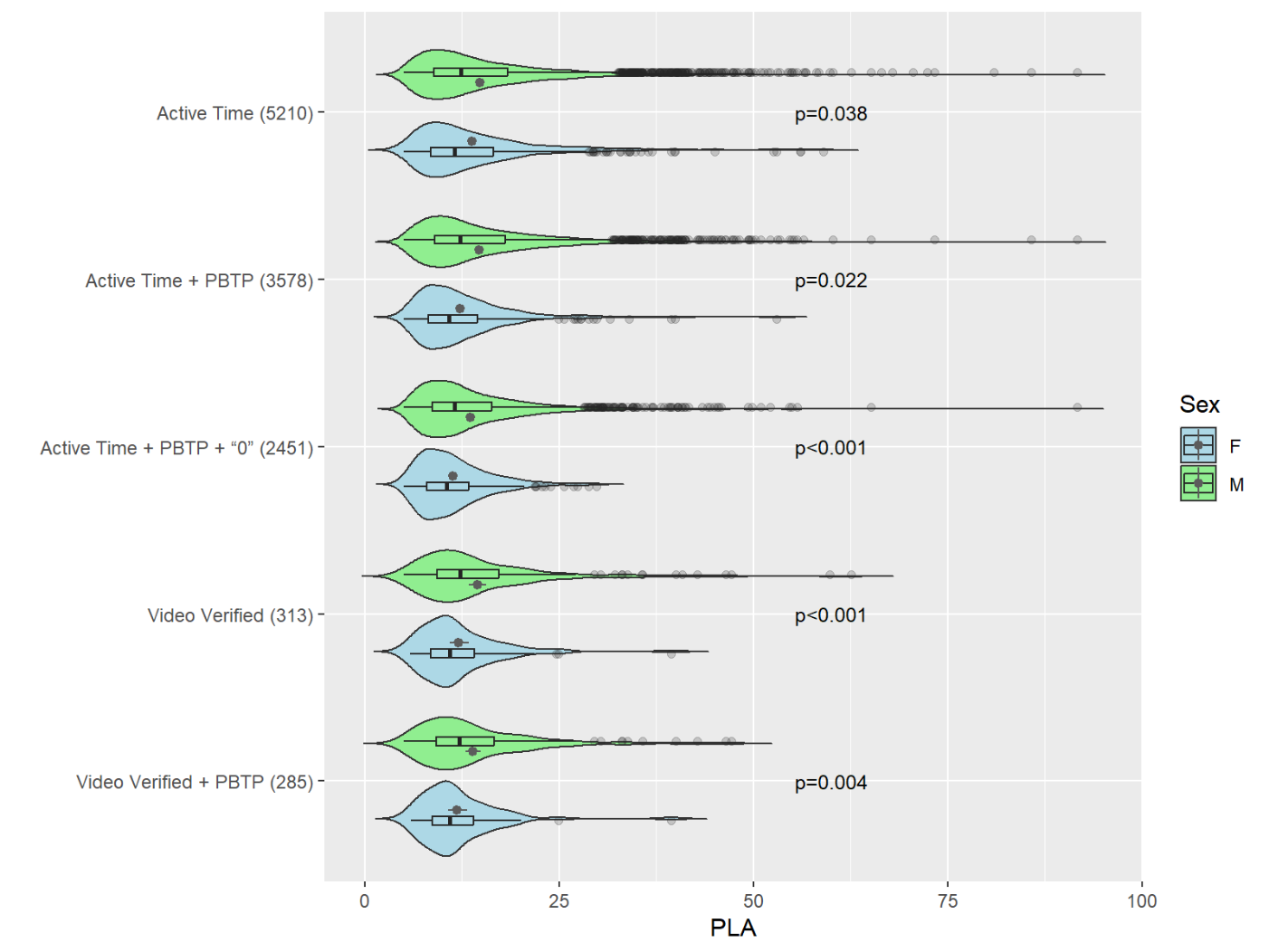


**Supplementary Figure 2**. Violin plots of Peak Linear Acceleration (PLA, g) between sexes. Boxplots within the violins represent the median and interquartile range; point with line represents the mean +/- standard deviation; p-value corresponds to the sex difference within data inclusion process.


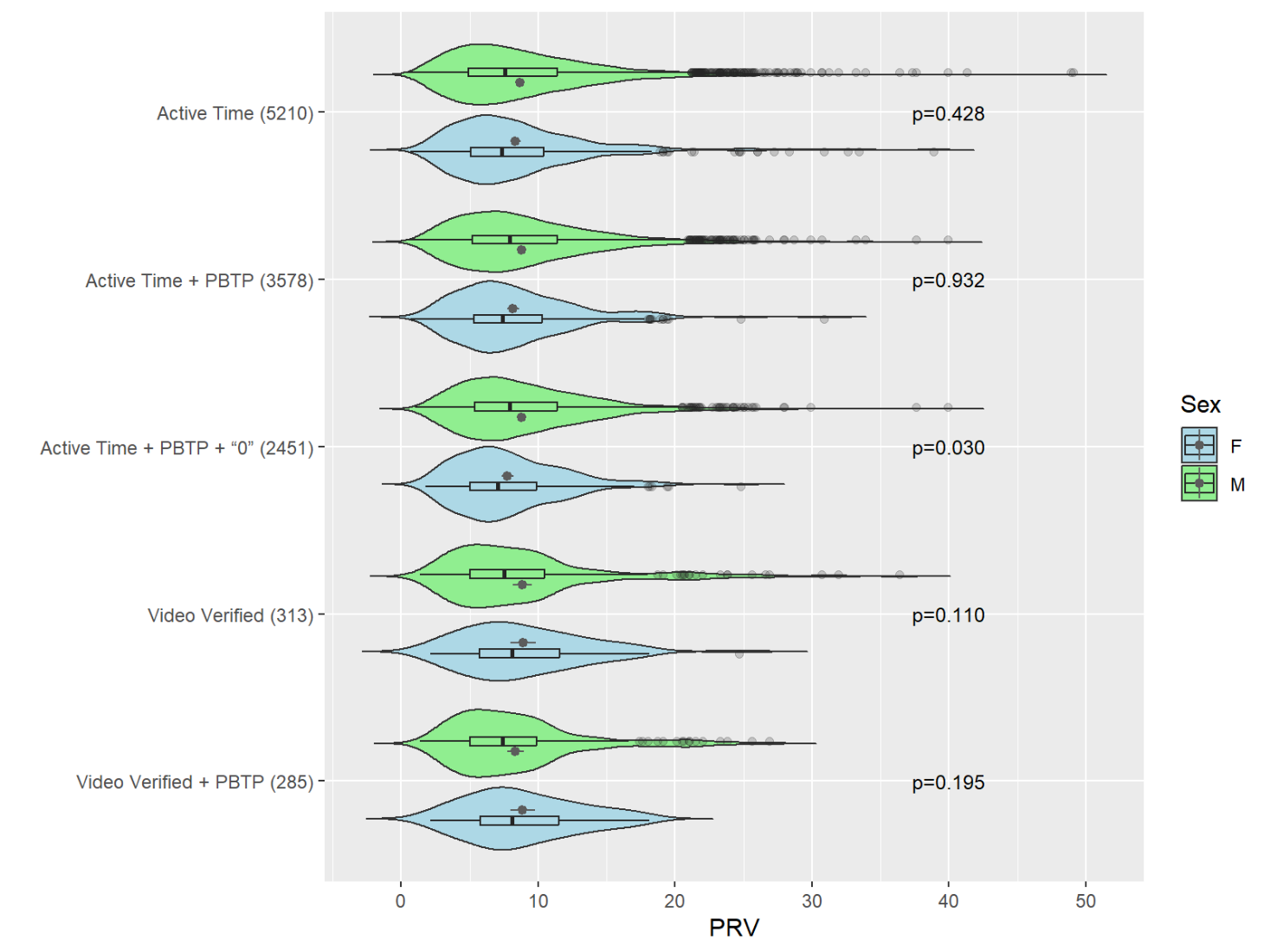
**Supplementary Figure 3.** Violin plots of Peak Rotational Velocity (PRV, rad/s) between sexes. Boxplots within the violins represent the median and interquartile range; point with line represents the mean +/- standard deviation; p-value corresponds to the sex difference within data inclusion process.
